# Social and Cultural Factors Affecting Uptake of Interventions for Malaria in Pregnancy in Africa: A Systematic Review of the Qualitative Research

**DOI:** 10.1371/journal.pone.0022452

**Published:** 2011-07-20

**Authors:** Christopher Pell, Lianne Straus, Erin V. W. Andrew, Arantza Meñaca, Robert Pool

**Affiliations:** 1 Centre de Recerca en Salut Internacional de Barcelona (CRESIB), Hospital Clínic-Universitat de Barcelona, Barcelona, Spain; 2 Centre for Global Health and Inequality, University of Amsterdam, Amsterdam, The Netherlands; Laboratory of Malaria Immunology and Vaccinology, United States of America

## Abstract

**Background:**

Malaria during pregnancy (MiP) results in adverse birth outcomes and poor maternal health. MiP-related morbidity and mortality is most pronounced in sub-Saharan Africa, where recommended MiP interventions include intermittent preventive treatment, insecticide-treated bednets and appropriate case management. Besides their clinical efficacy, the effectiveness of these interventions depends on the attitudes and behaviours of pregnant women and the wider community, which are shaped by social and cultural factors. Although these factors have been studied largely using quantitative methods, qualitative research also offers important insights. This article provides a comprehensive overview of qualitative research on social and cultural factors relevant to uptake of MiP interventions in sub-Saharan Africa.

**Methods and Findings:**

A systematic search strategy was employed: literature searches were undertaken in several databases (OVID SP, IS Web of Knowledge, MiP Consortium library). MiP-related original research, on social/cultural factors relevant to MiP interventions, in Africa, with findings derived from qualitative methods was included. Non-English language articles were excluded. A meta-ethnographic approach was taken to analysing and synthesizing findings. Thirty-seven studies were identified. Fourteen concentrated on MiP. Others focused on malaria treatment and prevention, antenatal care (ANC), anaemia during pregnancy or reproductive loss. Themes identified included concepts of malaria and risk in pregnancy, attitudes towards interventions, structural factors affecting delivery and uptake, and perceptions of ANC.

**Conclusions:**

Although malaria risk is associated with pregnancy, women's vulnerability is often considered less disease-specific and MiP interpreted in locally defined categories. Furthermore, local discourses and health workers' ideas and comments influence concerns about MiP interventions. Understandings of ANC, health worker-client interactions, household decision-making, gender relations, cost and distance to health facilities affect pregnant women's access to MiP interventions and lack of healthcare infrastructure limits provision of interventions. Further qualitative research is however required: many studies were principally descriptive and an in-depth comparative approach is recommended.

## Introduction

Malaria during pregnancy (MiP) is a major global health concern, resulting in adverse birth outcomes and poor maternal health [1]. In 2007, worldwide, over 125 million pregnancies occurred in areas of malaria transmission [2]. However, the morbidity and mortality caused by MiP is most pronounced in endemic regions of sub-Saharan Africa [3]. MiP compounds or provokes maternal anaemia, which when severe, increases the risk of maternal death: it has been estimated that malaria-related anaemia causes around 10 000 maternal deaths annually [4]. MiP can also result in low birth weight (estimated to cause around 100 000 infant deaths in Africa [4]), pre-term delivery, congenital infection and reproductive loss [3].

In spite of its significance for maternal and infant health, MiP has been viewed as a neglected area of study [5]. The MiP Consortium was therefore established to address the need for coordinated research on MiP. This global initiative, made up of 47 institutions, is undertaking a five-year programme of research (2007–2012) to evaluate new and to improve existing interventions for the prevention and treatment of MiP [6]. This review is part of the social science component of the consortium's Public Health Impact theme.

The suitability of MiP interventions depends primarily on the local disease context and prevailing levels of drug resistance. Current recommended malaria prevention for pregnant women in areas of stable moderate to high transmission include the administration, after quickening, of at least two doses of intermittent preventive treatment (IPTp) as part of routine antenatal care (ANC) [7]). In spite of questions posed by increasing resistance and other concerns, due to the lack of available efficacy and cost effectiveness data for other drugs and its consistent effectiveness [8], Sulfadoxine pyrimethine (SP) is currently used for IPTp in many sub-Saharan African countries [9]. The WHO also recommends that pregnant women receive insecticide treated bednets (ITNs), preferably long-lasting insecticide-treated nets (LLINs), as part of routine ANC, and that they are used throughout pregnancy [10]. Current recommended therapies for appropriate MiP case management include (for uncomplicated falciparum malaria) quinine/artesunate plus clindamycin and locally effective artemisim combination therapies (ACTs) [11]. The most appropriate treatment however depends on gestational age, the malaria species and severity, local patterns of drug resistance and drug availability [12].

Coverage targets set for MiP interventions have commonly not been met and challenges are yet to be overcome to ensure their effectiveness. The Roll Back Malaria “Abuja” target set in 2000 aimed for 60% coverage of recommended MiP prevention and treatment by 2005 [13] and was subsequently increased to 80% by 2010 [14]. However, data from 2004 to 2009 suggests that, in spite of national policies for prevention and control of MiP (and the limitations and scarcity of available nationally representative survey data), insufficient progress has been made towards the targets for coverage of IPTp and ITN use during pregnancy: 25% of pregnant women received at least one dose of IPTp and, overall, coverage was lowest in areas of highest malaria transmission [15].

The effectiveness of MiP interventions, as is the case of all health interventions, not only relies on their clinical efficacy, but also on a range of factors, such as the attitudes and behaviours of target groups, and of the wider community (including those implementing interventions) [16]. Attitudes and behaviours towards interventions are often shaped by social and cultural factors and such factors are particularly relevant to the demand for and supply of MiP interventions: attitudes towards and understandings of pregnancy, pregnancy care, malaria and other illnesses can interact and influence how, where and when pregnant women seek malaria prevention and treatment. Furthermore, the social and cultural context has important implications for the provision of malaria prevention or treatment, whether as part of routine ANC at a health facility or care sought from a local healer [17].

Data on knowledge and perceptions of MiP and the response to MiP interventions have generally been collected using (more) quantitative methods, such as questionnaires, with comparatively little research based on (more) qualitative methods (for example, in-depth interviews, focus group discussions or participant observation) focusing on MiP [5], [17], [18], [19]. Examples of findings from questionnaire-based research include: women's limited knowledge about the health consequences of MiP [20]; ANC health workers' lack of familiarity with IPTp [21], [22]; and the association of sleeping under a bed net when pregnant [23], [24], [25], [26] or receiving IPTp [27] with education and/or wealth. However, qualitative research that situates behaviour in its context is also necessary in order to give insight into complexities highlighted by quantitative research. For example, surveys in Malawi and Kenya have revealed high ANC attendance (88% and 74% respectively), yet a far lower proportion of women receive two doses of IPTp with SP (44% and 22% respectively) [27], [28]. In this case, a qualitative approach could supplement quantitative research by exploring the factors that affect uptake of MiP interventions even when pregnant women reach the clinic for ANC. Qualitative research is therefore an important tool to inform policies aimed at meeting the updated Abuja goals for recommended MiP prevention and treatment [14].

Reviews of the social science literature on malaria control and prevention in sub-Saharan Africa have revealed the increasingly important role of the social sciences in the study of behaviours related to the diagnosis, treatment and prevention of malaria in sub-Saharan Africa [18], [29], [30]. Yet the reviews also identify significant research gaps and a lack of translation of findings, particularly from qualitative research, into policy [18], [19], [29]. These reviews are not however focused on pregnant women; the only review of the social science literature on MiP relied on personal fieldwork experience and a non-systematic search strategy to develop models for qualitative approaches to research on factors affecting MiP [17]. To the authors' knowledge, no systematic review of the qualitative literature on MiP in sub-Saharan Africa has yet been published.

This article aims to provide a comprehensive overview of qualitative research on the social and cultural factors relevant to the uptake of MiP interventions in sub-Saharan Africa. Specific objectives include the identification of qualitative studies using a systematic search strategy, the description of the current state of qualitative research, and, using a meta-ethnographic approach, the synthesis of study findings and their organization along relevant themes identified from the studies. This will enable the identification of gaps in the qualitative research, the discovery of complementary or contradictory findings and the development of priorities for further research. The review is restricted to sub-Saharan Africa, because, as a result of epidemiological and structural factors, this is the region where MiP-related morbidity and mortality is most pronounced [3]. The review focuses on social science research based on qualitative methods to ensure the synthesis of findings is manageable, to draw attention to a neglected body of research and to identify the gaps in qualitative research. For practical reasons the review is restricted to English language literature.

## Methods

### Identifying relevant literature

Literature was identified through searching a range of databases. Searches were undertaken using OVID SP [31] and ISI Web of Knowledge [32] (see Appendix S1 for the complete list of databases). Searches were also carried out using CSA ILLUMINA ASSIA (Applied Social Sciences Index and Abstracts), CINAHL (the Cumulative Index to Nursing and Allied Health Literature) and The Cochrane Library. An additional search was undertaken using the MiP Consortium online library; searching this comprehensive bibliographic database that includes published and unpublished MiP-related literature [33] (most recent update, at time of search, 17^th^ April 2010) ensured that MiP-related research that was not accessible through conventional databases was included. The search terms used are detailed in Table 1. Test searches were carried beforehand to refine the search terms and ensure that relevant studies were captured.

**Table 1:** Search terms used and databases accessed.

	OVID SP, ISI Web of Knowledge†, ASSIA, CINAHL, and The Cochrane Library		MiP Consortium Library
	Africa OR African OR Angola OR Benin OR Botswana OR Burkina Faso OR Burundi OR Cameroon OR Cape Verde OR Central African Republic OR Chad OR Comoros OR Congo OR Cote d'Ivoire OR Djibouti OR Eritrea OR Ethiopia OR Gabon OR Gambia OR Ghana OR Guinea OR Kenya OR Lesotho OR Liberia OR Madagascar OR Malawi OR Mali OR Mauritania OR Mauritius OR Mozambique OR Namibia OR Niger OR Nigeria OR Rwanda OR Sao Tome OR Principe OR Senegal OR Seychelles OR Sierra Leone OR Somalia OR South Africa OR Sudan OR Swaziland OR Tanzania OR Togo OR Uganda OR Zambia OR Zimbabwe) NOT (African-American OR African American		qualitative OR sociolog* OR ethnograph* OR anthropolog* OR narrative OR focus group* OR interview* OR perception* OR belief* OR attitude*
AND	pregnan* OR maternity	AND	Africa
AND	qualitative OR sociolog* OR ethnograph* OR anthropolog* OR narrative OR focus group* OR interview* OR perception* OR belief* OR attitude*		
AND	Malaria		

† The ISI search was separated into two searches because these databases do not permit the inclusion of more than 50 Boolean operations in a single search.

**Figure 1:**
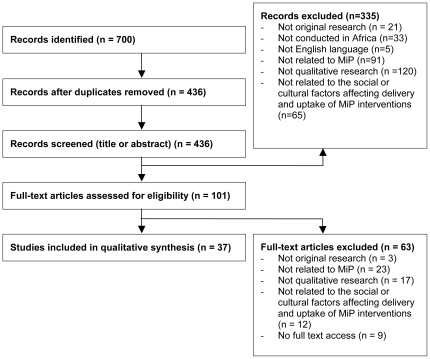
Summary of the article inclusion/exclusion process.

Citations and abstracts were downloaded into Endnote X2 and duplicates identified and deleted. All titles and abstracts were read and the following inclusion criteria were applied: original research; relating to MiP; about social or cultural factors relevant to the uptake of MiP interventions; conducted in an African site; employed qualitative methods and findings were derived from qualitative methods; and English language. Methods considered to be qualitative were interviews, focus group discussions, observations (including participant observation), ethnography and participatory methods (including free-listing, mapping and identification exercises). Access to the full text of the remaining articles was sought and the articles read and the inclusion criteria applied again. The full text of nine articles could not be accessed. Details of these articles can be found in Appendix S2. (See Figure 1 for a review of the article inclusion/exclusion process).

For articles that, in addition to relevant data, presented data that were unconnected to the uptake of MiP interventions, findings based these data were excluded from this review. If an article reported findings based on qualitative and quantitative methods, only findings based on the qualitative data were reviewed.

### Analysis

A meta-ethnographic [34] approach was taken to analyzing and synthesizing the qualitative findings from the included articles. Meta-ethnography provides an alternative approach to traditional narrative reviews and is increasingly used for the synthesis of qualitative findings, for which the techniques of meta-analysis, applied in reviews of quantitative research, are not appropriate [35], [36], [37]. A meta-ethnography endeavours to go beyond individual studies' accounts of phenomena, producing novel insights, yet also seeks to preserve the detail of the original studies [34]. In the first instance, included articles were read by two authors and initial data retrieved and tabulated (including the articles' aim, methods, participants and location, see Appendix S3). Articles were classified according to their main topic and location of data collection (see Table 2). Key themes and concepts were then identified from the articles. These key themes and concepts were used for the reciprocal translation of the studies, whereby they were mapped across the other articles in order to understand how the articles relate to one another (see Tables 3, 4, 5). The quality of the articles was independently assessed by two reviewers using a standardized grading scheme [38]. Ten areas (title and abstract; introduction and aims; method and data; sampling; data analysis; ethics; bias; results; transferability or generalizability; and implications and usefulness) were awarded grades ranging from one (very poor) to four (good), providing a maximum score of 40. In cases of grading discrepancy, the two reviewers discussed the scores and when an exact score could not be agreed on, a mean was taken of the two scores.

**Table 2:** Main topics of the articles reviewed.

Main focus of the article	Region			Total
	West Africa	East Africa	Southern Africa	
MiP treatment or prevention	4	8	2	14
Malaria treatment or prevention	4	12	1	17
ANC service use		4		4
Anaemia during pregnancy	1*			1
Reproductive loss			1	1
**Total**	9	24	4	37

*One article, included in MiP, focused equally on malaria and anaemia during pregnancy.

**Table 3:** Mapping of key themes and concepts across the reviewed articles: illnesses and vulnerability.

Region	Country	Year	Reference	Disease categories corresponding to malaria or MiP	Explaining malaria or MiP	Vulnerability of pregnant women (including adolescents)	Seriousness of MiP	Anaemia
East Africa	Ethiopia	2004	[48]	*Busaa* – mild and severe.	Lack of food and poor hygiene.	More vulnerable to malaria.	Requires prompt action.	
	Kenya	2005	[69]				Viewed as a health problem.	
		2010	[49]			More vulnerable to malaria.		
	Tanzania	1996	[40]	*Homa ya malaria*, not associated with malaria or anaemia during pregnancy. Disease of outsiders.			MiP-related problems explained in other ways. Fever normal.	
		2005	[58]	Several terms used including “homa” (fever).			Causes still births, congenital malaria, excessive bleeding pre-/post-partum.	In pregnant women linked to malaria.
	Uganda	1994	[39]	*Omusujia, -* any kind of fever or feelings of illness.	Mosquito bits, dietary and environmental factors.	No more vulnerable to malaria than other groups.		
		2001	[52]			More vulnerable to malaria.	Minority linked MiP with miscarriages.	
		2006	[55]	Mild and severe malaria distinguished.		More vulnerable to malaria, but not more so adolescents.	Fever normal after delivery.	
		2006	[57]			At high risk of malaria. Adolescents not especially vulnerable to MiP but occupy precarious social position.		
		2006	[43]	*Omusujia* represents all types of fever.	Mosquito bites, poverty, dirty water, poor hygiene and “lack of blood”.	Pregnant women (and children) most vulnerable to malaria. Adolescent not at special risk of MiP.	Main cause of illhealth for pregnant women. Can result in miscarriage.	Attributed to diet.
		2007	[66]			Adolescents not especially vulnerable to malaria.		
		2008	[53]			Pregnant women (and children) most vulnerable to malaria.		
Southern Africa	Malawi	2006	[42]	*Malungo*, implied a common and fairly harmless illness.	Mosquito bites and hard work.	Vulnerable to witchcraft.	Not viewed as more serious than malaria. Fever normal.	
		2007	[56]			Vulnerable to witchcraft.		
		2008	[45]	One of “modern” diseases.			MiP causes pre-term birth.	Causes pre-term birth.
	Zambia	2001	[54]			More vulnerable to malaria.		
West Africa	Burkina Faso	2002	[44]	No one-to-one equivalent – *soumaya* used instead.	Humidity, rain and cold.			
		2004	[54]					Women unsure how to prevent anaemia (and malaria)
	Ghana	1994	[47]	*Fever* (and *asra* in children).	Mosquito bites, heat and poor hygiene.			
		1997	[60]					Due to dietary factors.
		2007	[46]	“Male” fever or ordinary fever and hard to cure.	Mosquito bites, heat, poor hygiene and evil spirits.			Consequence of MiP.
	Nigeria	2001	[50]			More vulnerable to malaria.		
	Senegal	2001	[51]			More vulnerable to malaria.		
	The Gambia	2009	[59]				MiP causes pre-term birth and affects foetus.	Caused by MiP.

**Table 4:** Mapping of key themes and concepts across the reviewed articles: interventions.

Region	Country	Year	Reference	ITNs	IPTp/SP/Chemopraxis/IST	Case management
East Africa	Ethiopia	1996	[62]		Chemopraxis non-compliance due to lack of knowledge; safety fears; lack of time; distance to clinic.	
		2004	[48]	Lack of confidence in ITNs.	SP perceived to be less effective.	
	Kenya	2005	[69]		Women were aware of dangers of not taking IPTp.	Women preferred “Western” drugs, refused admission for severe malaria to avoid leaving children unsupervised, bought drugs over-the-counter and self-medicated.
		2010	[49]	Affordability a problem. White ITNs preferred. Suspicions about targeting of dissemination to women and children. Husbands controlled finances and influenced purchase of ITNs.		
	Sudan	2004	[75]			Fee exemption fee enabled women to purchase a full course of treatment.
		2008	[63]	Demand for ITNs but lack of knowledge about insecticide treatment and concerns about net sizes. Used only after rainy season.		
	Tanzania	2003	[74]	Pregnancy women were aware of ITNs but awareness of how to take part in voucher scheme low.		
		2005	[58]		Feared side effects (including miscarriages) women so did not swallow IPTp tablets. Problems of delivery: lack of water and cups. Not all staff understood IPTp.	To treat malaria women used formal healthcare services, self-medicated and sought the help of traditional healers.
		2006	[73]	Despite information at ANC clinic, not all women knew about ITN voucher scheme.		
		2008	[41]		Coverage, especially of 2^nd^ IPTp dose, was low. Problems with: supply of SP, DOT, skills and knowledge of ANC staff, and reporting system.	
		2008	[68]		Negative views of SP during pregnancy included side effects and large babies (complicating delivery).	
		2009	[71]	ITNs viewed as a positive aspect of formal ANC.	SP viewed as a positive part of formal ANC.	
		2010	[64]	National ITN voucher scheme encouraged ANC attendance. Concerns about distance to retailers.		
	Uganda	1998	[67]			Cholorquine perceived as causing itching that deterred use. Bitter drugs should not be taken during pregnancy. Formal health system last resort for treating malaria.
		2001	[52]	Concerns of effects of insecticide treatment for ITNs on foetus and mother.		
		2006	[55]		SP viewed as a cure (not prevention) and strong enough to cause abortions or foetal abnormalities. Health workers promoted idea of strength and encouraged women to drink sweet liquids they could not afford. IPTp viewed as promoting resistance to SP.	
		2006	[57]	ITNs not used because of their price and perceived dangers – influencing pregnancy outcomes. Women reluctant to buy ITN because this required money and if they spent money their husband would accuse them of have an affair.		
		2006	[43]		IPTp relatively unknown.	
		2007	[66]	New delivery approach led to increased use of ITNs.	Women accepted IPTp due previous experience of MiP and because they trusted drug vendors and TBAs providing SP.	
		2008	[53]			Medication kept for emergencies. Self-treatment common till symptoms worsened. Price main consideration.
		2008	[70]		Women frustrated at “only” receiving SP at ANC	
		2010	[72]		IPTp delivered but due to range of problems not done so in an integrated manner with HIV/AIDS prevention.	
Southern Africa	Malawi	2007	[56]		Unclear messages re timing of IPTp. SP shortages. ANC staff had little knowledge of SP. Women took SP as trusted health staff. Women viewed SP as treatment not prevention.	To treat malaria women buy over-the-counter drugs and self medicate.
		2008	[45]		Bitter medicine, including SP thought to cause miscarriages and still births. But taken if prescribed.	Women buy medication, including SP, from vendors, in spite of advise to go to ANC clinic.
	Zambia	2001	[54]	ITNs identified as harmful to pregnant women.		
West Africa	Burkina Faso	2002	[44]	ITNs used.	Cholorquine reported as a way of preventing malaria.	
	Ghana	1994	[47]			Pregnant women seek treatment for malaria at the clinic more readily than other groups. Medication in early pregnancy not advised.
		2007	[46]	ITNs not used as there was scepticism about possibility of preventing malaria.		Women always sought advice from experts (health workers or traditional healers) before taking medication.
		2010	[65]	ITN a motive for attending ANC.	IST unnoticed as different from IPTp. Blood tests were accepted provided purpose was given.	
	Nigeria	2001	[50]	Concerns about insecticide treatment for ITNs, with fumes possibly causing miscarriage.		
	Senegal	2001	[51]	Concerns about insecticide treatment for ITNs, linking smell to damage to the foetus.		
	The Gambia	2009	[59]		Confusion about IPTp and iron treatment and ignorance of the IPTp schedule.	Women unclear about the drugs given in ANC but accepted them as safe if given by health workers. Cholorquine avoided in pregnancy due to bitter taste.

**Table 5:** Mapping of key themes and concepts across the reviewed articles: ANC services.

Region	Country	Year	Reference	Perceptions of ANC services	Structural factors affecting access to and delivery of ANC services
East Africa	Tanzania	2005	[58]	Complaints about lack of laboratory services, services, shortages of staff and the behaviour of staff contributed to negative opinions of ANC services at the clinics.	Lack of resources at clinic.
		2009	[71]	Obtaining the “maternity” card, having the position of the baby checked motivated women to attend a health facility for ANC. They had also heard of the “malaria injection”, SP tablets, ITN vouchers and the blood test for tetanus. General positive view of ANC services.	
		2010	[64]		Complaints about varied user fees, penalties and punishments for late attendance, and unnecessary referrals, which also incurred costs. Time required to travel the distance to the clinic and waiting for services also influenced access.
	Uganda	1998	[67]	Obtaining the “maternity” card and checking the position of the baby motivated women to attend a health facility for ANC. Complaints about costs, lack of staff, poor examinations.	Cost described as an issue.
		2006	[55]	Obtaining the “maternity” card motivated women to attend a health facility for ANC. Women did not view ANC clinic as offering disease prevention, but attended ANC services when ill and complained about lack of drugs. Health workers advised pregnant women to drink sweet fluids when taking SP as IPTp deterring women from the clinic, as they could not afford these liquids. Complaints about rudeness of staff.	Distance to the clinic and cost of services influenced demand for ANC services. Husband control financial resources so women dependent on husbands to access ANC services.
		2006	[57]	Obtaining the “maternity” card motivated women to attend a health facility for ANC. Fear of rebukes from health staff regarding dress, lateness, use of herbal treatments.	Distance to the clinic and cost of services influenced demand for ANC services.
		2006	[43]	Obtaining the “maternity” card motivated women to attend a health facility for ANC. As did receiving tetanus immunization, testing for anaemia and being tested for other diseases.	
		2007	[66]	Health workers reported that women lack knowledge about dangers of MiP and for this reason do not attend ANC.	Distance to the clinic, cost of services, and stock outs influenced demand for ANC services.
		2008	[70]	Obtaining the “maternity” card motivated women to attend a health facility for ANC. ANC literally referred to as “drinking medicine” and associated with being ill. Women attend ANC when ill and expect to receive medication.	Financial constraints limit access to ANC services.
Southern Africa	Malawi	2007	[56]	Obtaining the “maternity” card and ensuring the baby was growing well motivated women to attend a health facility for ANC. Women delayed formal ANC because revealing an early-stage pregnancy put them at risk from witchcraft and animosity from the community, and in a setting where miscarriages were common, they wanted to be certain about the pregnancy before making the journey to the clinic.	
		2008	[45]	Rebukes from health workers discouraged women from attending ANC.	Transport costs influence access to ANC services.
West Africa	Burkina Faso	2004	[61]	Satisfied with ANC service quality. A small proportion knew services were free. ANC therefore seen as costly.	
	Ghana	2010	[65]	Obtaining the “maternity” card and an ITN motivated women to attend a health facility for ANC. Women unclear about specific diseases being treated or prevented during ANC visits. Checking the position of the pregnancy was another motivating factor. Health workers treated the pregnant women well and women trusted health workers.	

## Results

### The nature of the evidence

Of the 37 studies reviewed, 14 focused on MiP, its treatment or prevention. The remaining studies dealt tangentially with MiP, as part of the broader topic of malaria treatment and prevention, factors related to ANC service use, anaemia during pregnancy or reproductive loss (see Table 2 for details).

The majority of the studies were carried out in East Africa (24 of 37). The geographical bias is largely explained by the number of studies that have been undertaken by the same or similar research teams: six of the articles from Uganda have the same lead author (and several of these are based on the same data) and three of the studies from Tanzania have the same lead author. Nevertheless, there is a paucity of research from southern and central Africa, and only three studies from francophone countries.

The studies relied largely on individual (33) and group interviews (31) and a minority of studies (7) utilized qualitative data from observational methods, including participant observation. Twenty-two studies employed quantitative data collection techniques (questionnaire-based surveys) in addition to the qualitative methods and six of the nine articles that employed only one type of qualitative methods also collected data using quantitative methods. A variety of respondent types participated in the studies: pregnant women, community members, opinion leaders, traditional birth attendants (TBAs), health workers and policy makers. However, of the 37 articles reviewed, only 17 included pregnant women as a specific group of respondents.

Despite the overall paucity of qualitative research on MiP in sub-Saharan Africa, as can be seen in Table 6, during the last 20 years there has been an increase in studies, most markedly over the last five years.

**Table 6:** Year of publication of the articles reviewed.

Year published	N
2006–2010 (May)	21
2001–2005	10
1996–2000	4
1991–1995	2
-1990	0

Based on the standardized grading scale [38], the reviewed studies were, on average, of fair quality (mean score 30.2/40 N = 31). The formats (study reports or theses) of six studies prevented their grading. Two studies from the 1990s received the lowest score of 25/40 [39], [40] and one study from 2008 received the highest score of 35/40 [41] (for a complete list of the grading scores see Appendix S3). The studies, particularly earlier studies, lacked details of any efforts undertaken to minimize bias and of ethical issues. The explanations of sampling procedures utilized were also limited.

### Qualitative synthesis of findings

Based on the organization of the articles and their findings, the synthesis of qualitative findings is divided into several topics that influence the uptake of MiP interventions: concepts of malaria and risk in pregnancy; attitudes towards malaria prevention and treatment; perceptions of ANC services; and structural factors affecting the delivery and uptake of MiP interventions. As the principal MiP interventions are delivered as part of ANC services, their success will depend on appropriate ANC attendance and therefore factors affecting ANC service use are included in the synthesis.

#### Concepts of malaria and risk in pregnancy

Several studies confirmed the common observation that local disease categories often do not correspond to biomedical definitions of malaria (and MiP). In rural southern Malawi the term used to describe malaria, *malungo*, implied a common and fairly harmless illness [42]. In south-western Uganda, *omusujia,* the word used to translate malaria, referred to any kind of fever or feelings of illness [39]. And Mbonye and colleagues [43] also described how, in central Uganda, through a blurring of disease categories, *omusujia* represented all types of fever. Okrah and colleagues [44] reported that in north-western Burkina Faso no “one-to-one equivalent of the biomedical concept of malaria” existed in any of the local languages. The broad term *soumaya* was therefore used in malaria related public health discourse, yet this condition encompassed a very wide range of symptoms [44]. On the coastal plain of Tanzania, respondents talked about *their* diseases and the diseases of outsiders (referring to foreign and Tanzanian internal migrants). The term linguistically closest to malaria and that used for public health education, *homa ya malaria*, was considered to represent a disease of outsiders. Moreover, *homa ya malaria* was not actually associated with malaria or anaemia during pregnancy [40]. Participants in Tolhurst and colleagues' study in southern Malawi attributed pre-term delivery to both “modern” and “traditional” illnesses, of which malaria was categorized as “modern” [45].

Local disease categories for fever and malaria can have implications for treatment or prevention seeking behaviour. For example, respondents from two areas in southern Ghana divided malaria into two types of fever, ‘common’ fever and ‘male’ fever; the former was relatively easy to cure with herbs at home, whereas the latter required attention from a health provider [46]. In Tanzania, the internal migrants, who used the “outsider” disease categories, which included *homa ya malaria* (malaria fever), were reported to show higher levels of ITN ownership and re-treatment [40].

Respondents from a number of study sites associated mosquito bites with malaria (or the local disease category that included malaria) (e.g. [39], [42], [43], [47]). However, in several settings, other factors were viewed as playing a role: in south-central Ethiopia, a lack of food during the fasting season and unhygienic practices were linked to malaria infection (or *busaa* as it was locally termed) [48]; in southern Ghana, exposure to heat, poor hygiene and evil spirits were important factors [46], [47]; Kengeya-Kayondo and colleagues also reported that in southwestern Uganda *omusujia* could be caused by dietary or environmental factors [39]; whereas in central Uganda, poverty, dirty water, poor hygiene and a lack of blood contributed to *omusuji* [43]; in southern Malawi, *malungo*, could be caused by hard work [42]; and in Burkina Faso humidity, rain and cold also reportedly led to *soumaya* [44].

Respondents in several countries (Kenya [49], Ethiopia [48], Nigeria [50], Senegal [51], Uganda [52], [53] and Zambia [54]) viewed pregnant women as particularly vulnerable to malaria infection, yet less vulnerable than children. However, in one study from south-western Uganda, malaria was identified as universal amongst all age groups and pregnant women were not singled out [39]. Although few studies subdivided pregnant women by age, age was contemplated in one study carried out in central Uganda: the study's findings suggest that respondents did not recognize pregnant adolescents as a group at particular risk of malaria [55].

Several studies emphasized that pregnant women's vulnerability to malaria should be contextualized among other risks and dangers inherent to pregnancy. In southern Malawi for example, pregnancy itself was seen as a vulnerable state in which women are at greater risk of illnesses generally, particularly harm caused by witchcraft [42], [56]. In central Uganda, Mbonye and colleagues emphasized the social vulnerability of adolescent pregnancy. Adolescents did not access healthcare because of the shame attached to pregnancy and commonly tried to terminate their pregnancy by any means possible [57].

Differences in perceived seriousness of MiP and the relationship between perceived seriousness and behaviour were identified in the studies. In southern Malawi, pregnant women did not view MiP as serious, or any more serious than malaria outside of pregnancy [42]. Similarly, in coastal Tanzania, MiP was not seen as a concern and problems that medically could be attributed to MiP such as anaemia, headaches and infant mortality were attributed to different causes [40]. Several studies found that fever is considered normal during pregnancy and post-partum [40], [42], [43], [57]. In contrast, respondents from studies carried out in Uganda [39], [43], [57], in north-eastern Tanzania [58], in south-central Ethiopia [48], in The Gambia [59] and (a different study) in southern Malawi [45] identified MiP as serious and associated it with miscarriages, pre-term birth, swelling, dizziness, high blood pressure, abortion, dehydration, headache, vomiting, persistent menstruation, weakness and joint pain.

Four studies reported perceptions about the link between malaria and anaemia in pregnancy. MiP was not recognized as a cause of anaemia (characterized locally as a lack of blood) amongst adolescents on Ghana's coastal savannah, which was rather seen as due to dietary factors and anxieties (particularly for unmarried girls) [60]. Central Ugandan respondents attributed anaemia (also “lack of blood”) in pregnancy to diet [43]. In The Gambia [59] and in a later study in the same region of Ghana [46] anaemia was however described as a consequence of MiP. The authors of the more recent study in the same region of Ghana suggest that this change in understanding of MiP and anaemia over time may be due campaigns of health education [46].

#### Attitudes towards malaria prevention and treatment

Even when MiP is perceived as a serious risk or illness, attitudes towards particular interventions or ideas about malaria prevention in general may influence the uptake of preventive measures. For example, in Burkina Faso, pregnant women were reportedly unaware of how to prevent malaria (and anaemia) [61], and in southern Ghana, although MiP was recognized as having important health consequences, malaria prevention was not seen as possible and this impacted upon the use of preventive measures [46]; whereas in south-western Uganda, dietary measures were seen as the main means of preventing malaria [39]. Many women in Sudan did not comply with chloroquine chemoprophylaxis, as recommended in the 1990s, due to a lack of knowledge about its benefits and fears about the safety of taking chloroquine in pregnancy [62].

Low bednet usage was attributed to a range of factors: seasonality of use and complaints about nets being uncomfortable or not of a suitable size [57], [63]; perceptions surrounding ITNs' “chemicals”, which were viewed by some respondents as harmful to pregnant women and unborn children [57]; concerns related to the retreating of ITNs and the effects of the insecticides upon pregnant women and the unborn child [50], [51], [52], [54]; and affordability and availability [49], [57]. Also, in Kenya, respondents expressed concerns about the targeting of interventions, particularly the distribution of ITNs to specific population groups, mainly children and pregnant women; their anxieties were framed in terms of comments about attacks on Kenyan's fertility [49]. There was however evidence of demand for ITNs. In coastal and highland areas of Tanzania, Mubuyazi and colleagues reported that the national ITN voucher scheme for pregnant women had encouraged women to attend ANC at a clinic and collect their voucher [64]. Obtaining an ITN was also a motive for women in central Ghana to attend a health facility for ANC [65].

Respondents were often unsure about drugs used for IPTp and whether the drugs prevented MiP. In spite of this uncertainty, in two studies the trust placed in those administering IPTp provoked adherence. In The Gambia, respondents were unclear about the drugs used for IPTp, but they accepted the drugs as safe because health workers administered them [59]. Women in Uganda were also willing to take IPTp because they trusted TBAs and drug vendors who recommended IPTp[66]. SP was perceived to be a good cure by respondents in central Uganda however they showed little awareness of SP being used to prevent MiP [55]. Similarly, in southern Malawi, SP was associated with treating rather than preventing MiP and respondents reported that if a woman was issued SP at the ANC clinic, it was because she was sick [56].

In contrast, respondents from several studies viewed SP as harmful, suggesting that its “strength” caused miscarriages [42], [55], [58], [67], [68] and describing side effects that included mouth sores, fatigue, fever, rashes and itchiness [67], [68]. In north-eastern Tanzania, Mubyazi and colleagues reported similar concerns deterring women from taking SP as IPTp [58] and Mushi and colleagues described how women linked taking SP with large babies and therefore difficult deliveries, which they were keen to avoid [68]. However, these studies suggested that although these perceptions exist, there were very few cases of adverse effects, and that these ideas were based on hearsay rather than personal experience [58], [68].

One recent study that explored a potential alternative to IPTp, intermittent screening and treatment (IST), found that pregnant women did not distinguish between the two strategies and did not complain about the blood test required for IST [65]; the differences between the two strategies were diluted by the other ANC procedures.

The reviewed studies revealed diverse attitudes towards malaria medication and a range of sources of treatment for malaria treatment during pregnancy. In Ghana, self-medication was seemingly infrequent, as pregnant women would not take medicines unless advised by an “expert” – a traditional healer or a health worker [46]. Previous research in Ghana suggested that pregnant women were keen to care for their pregnancy and therefore sought formal treatment for malaria; this differed markedly from the behaviour of other adults who mainly self-medicated with herbal remedies and other medicines at home [47]. However, women were uncertain about taking any medication in early pregnancy [47]. In contrast, Kenyan pregnant women often bought drugs over the counter and self-treated: they preferred western pharmaceuticals and were familiar with their names [69]. Women from the same study identified MiP as an important health problem, yet they often refused to be admitted to hospital for treatment for severe malaria because they could not leave their children unsupervised [69]. In north-eastern Tanzania and western Uganda, to treat malaria, women accessed formal health services, self-medicated and sought the assistance of traditional healers or TBAs [58], [67].

As with IPTp, the perceived strength and side effects of treatments influence women's readiness to take them during pregnancy. In Uganda, Cholorquine's bitterness and the associated side effects (itching) discouraged women from taking it [67]. Mbonye and colleagues also reported concerns in Uganda about taking quinine during pregnancy [55]. Respondents in Malawi identified bitter medicines (including SP) as a cause of miscarriage and stillbirth, yet, in spite of this, they bought drugs (including SP) from drug sellers [45]. Waisa and colleagues also reported that diseases during pregnancy tend to be treated at first with herbs in the home prior to seeking assistance at the clinic [70].

#### Perceptions of ANC services

Many factors were identified as influencing whether women receive ANC in a health facility. Several studies identified obtaining the maternity (or health or ANC) card as a factor that motivated women to attend a health facility for ANC [55], [56], [57], [65], [67], [70], [71]. The cards were desirable because they enabled women to deliver at health facilities without recrimination from health workers. Acquiring an ITN also motivated some women in coastal and highland areas of Tanzania and central Ghana to access ANC [64], [65]. Being tested for sickness, checking the progress of the pregnancy and having one's stomach examined at the ANC clinic were described as other important motivating factors [57], [64], [65]. In Uganda, pregnant women did not view ANC clinics as offering disease prevention, but attended ANC services when ill [55], [70].

Although gestational age at first ANC visit has consequences for the provision of MiP interventions, only one of the reviewed studies surveyed factors that influence timing of first ANC visit. In southern Malawi, women preferred not to start attending ANC early in their pregnancy (before about 24 weeks); they delayed because revealing an early-stage pregnancy put them at risk from witchcraft and animosity from the community, and, in a setting where miscarriages were common, they wanted to be certain about the pregnancy before making the long journey to the clinic [56].

Once at the ANC clinic, how care is provided can also affect women's understanding of ANC services and of MiP interventions. In Malawi, in a multi-lingual setting, health education was mainly delivered in the nurses' mother tongue rather than that of pregnant women [56]. In Uganda, Mbonye and colleagues reported that health workers provided misleading information to women accessing ANC: they advised pregnant women to drink plenty of sweet fluids when taking SP as IPTp, “to help them withstand the strength of Fansidar” (the brand name for SP) [55]. As a consequence, women were deterred from visiting the clinic because they could not afford to buy these liquids [55]. Women in Malawi also reported that being rebuked by health workers discouraged them from attending ANC [45].

#### Structural factors affecting the delivery and uptake of MiP interventions

Overall, fifteen studies identified structural factors that can affect the delivery and uptake of MiP interventions, particularly those delivered through ANC services. Factors purely affecting demand for ANC services included distance to the clinic and cost of services [43], [55], [57], [58], [61], [66], [67]. Once at the ANC clinic, supply and demand were influenced by a range of factors: pregnant women and health workers complained about the lack of resources, including staff, laboratory tests, drugs, and clean cups and water to administer IPTp with SP [56], [58], [67]. Whilst Mubyazi and colleagues reported that in coastal and highland areas of Tanzania women were generally satisfied with ANC services, they also described the complex nature of ANC services and fees, which discourage some women: participants complained about varied user fees, penalties and punishments for late attendance, and unnecessary referrals, which also had to be paid for [64].

Tanzanian policy makers reported a range of factors that influence the delivery and uptake of IPTp. In addition to late ANC attendance, they reported concerns about compliance with the IPTp delivery guidelines at healthcare facilities, problems of monitoring delivery, a lack of SP, questions about the effectiveness of the direct observation treatment (DOT) procedure, and poor communication with the media, which led to misleading newspaper articles about the appropriateness of SP for IPTp [41]. One study in Uganda highlighted the difficulty of providing malaria and HIV prevention in an integrated manner due to insufficient drug stocks and health workers lacking adequate skills [72].

Various strategies have been employed to improve the delivery of MiP interventions: in central Uganda delivering IPTp in the community rather than at the ANC clinic improved uptake and increased awareness of the dangers of MiP [66]; and, in eastern Sudan, bednet distribution through local committees and leaders authorities encouraged their use more than distribution through the ministry of health [63]. In Tanzania, a social marketing approach, using vouchers that pregnant women received as part of ANC and then exchanged for a subsidized bed net, has been trialled. However, despite initial high voucher return rates, there have been mixed reports of awareness about the scheme, how to take part, and the intended recipients of the nets [73]. Even at a discounted price, some women were not able to afford the nets and nets also “leaked” from the system [74]. There was also disagreement amongst policy makers in Tanzania regarding the effectiveness of the subsidized system [41]. In Sudan, when exempted from costs, pregnant women visited the clinic more often and were able to buy a full course of medication [75]. The cost of malaria medication was also the priority for respondents from one study in Uganda [53].

Finally, household decision-making dynamics can also have an impact on how women access ANC and therefore MiP interventions. For example, in Uganda and Kenya, husbands were reportedly in control of domestic resources and acted as ultimate decision makers. They therefore influenced whether their wives sought ANC at a health facility or not and whether they purchased an ITN [49], [57].

## Discussion

The reviewed studies illustrate that the symptoms (particularly fever) or symptom complexes often brought about by malaria infection are associated with various locally defined illnesses [39], [40], [42], [43], [44]. Furthermore, although many respondents linked mosquitoes bites with malaria (or the corresponding local disease category), other factors were also viewed as important, such as exposure to heat, cold or rain, diet and hard work [39], [42], [43], [44], [47], [48]. Given that women experience malaria-like symptoms during pregnancy [42], particularly fever, understanding how these symptoms are interpreted in local disease categories (and what action is taken) is of particular public health interest and is essential for the appropriate design of strategies of health education focused on MiP.

How women understand their vulnerability to malaria and other illnesses when pregnant is key to designing and promoting appropriate MiP interventions. Study respondents often viewed pregnant women as particularly vulnerable to malaria infection and identified serious potential health impacts of MiP [48], [49], [50], [51], [52], [54]. However, the vulnerability of pregnant women was often described in more general terms [42], [56]. Moreover, although adolescents are at greater risk of MiP and its adverse effects [3], the social vulnerability of pregnant adolescents is also important; pregnant adolescents suffer stigma, which has implications for pregnancy care and requires further study in African contexts [57].

Although clinicians, policy makers and scientists may focus on reducing the burden of MiP, the reviewed research suggests that pregnancy care is often less illness specific; women act, within certain socio-economic, social, cultural and individual constraints, so as to protect themselves and their pregnancies from what they see as the most important threats. Some of these threats, such as those described by Tolhurst [45], are not perceived in biomedical disease categories, and some are given personalistic explanations related to witchcraft [76], [77]. The promotion of pregnancy care that is based on a more holistic understanding of illness, and less disease-focused, is more likely, compared with a disease-focused approach, to resonate with women's own ideas of protecting their pregnancy.

Antenatal care at health facilities is a key platform for the delivery of MiP interventions. Therefore factors that influence the timing of ANC visits impact MiP interventions. A prior literature review has shown that a woman's (and her husband's) education, cost (travel and upfront fees) and distance to the clinic are key determinates of ANC attendance [78]. A women's age is also important, albeit in a complex manner; although young women in some contexts hide their pregnancy and delay formal ANC, older women may also delay or avoid ANC because they are accustomed to pregnancy [78], [79]. Ideas about the purpose of ANC services influenced ANC attendance [55], [70], [80]: women viewed ANC as providing treatment rather than prevention, and only attended when ill. Health workers' attitudes and behaviour towards pregnant clients and attitudes towards specific services offered can also potentially deter women from accessing ANC at health facilities [55]. For example, fears about HIV-testing (or status disclosure) has been reported as influencing ANC attendance in rural Zimbabwe [79]. The factors influencing ANC attendance are not only complex, but also often context-specific, requiring locally appropriate strategies to improve attendance [76]. Chapman for example, demonstrates the value of long-term qualitative research for providing an in-depth understanding of pregnancy care [76]. However, there is a general lack of qualitative studies, which could inform locally appropriate strategies [78].

A range of factors influences how recommended policies for MiP prevention and care are implemented and received locally. In addition to issues of cost and accessibility, deterrents to ITNs use and IPTp uptake included dissatisfaction with and anxieties about ITNs or the insecticide re-treatment [50], [51], [52], [54], [55], [57] and concerns about the side effects of taking SP during pregnancy [45], [55], [58], [68]. Concerns about SP and other malaria drugs were linked to local discourses [58], [68] and reinforced [55] or dispelled [46], [59] by health staff. Exploring the social relationship between pregnant women and healthcare staff in the context of formal ANC, and the influence of local discourses is therefore key to understanding attitudes towards and uptake of malaria interventions.

Structural factors affect the uptake of MiP interventions at a range of levels: from household decision-making to national and international implementation strategies. The reality of ANC in health facilities complicated the implementation of national IPTp guidelines in Tanzania [41] and the delivery of integrated HIV and malaria prevention in Uganda [72]. And although other strategies for IPTp and ITN delivery demonstrated some success, there remain structural challenges linked to delivery (e.g. shortages at healthcare facilities and a lack of health worker training) and uptake (e.g. cost and distance) to overcome [41], [63], [66], [74]. Access to MiP interventions (and formal ANC) is also influenced by the dynamics of in-household decision-making, and gender relations [57]. The qualitative research methods available to social scientists are well suited to the study of structural factors across multiple levels enabling observed phenomena to be situated within their household, community, cultural, social, economic and historical context.

### Priorities for further research

To explore thoroughly the socio-cultural factors that affect the uptake of MiP interventions, malaria must be contextualized within local understandings of illness, (social and economic) vulnerability and care during pregnancy. However, many of the reviewed studies were disease-focused and the authors were unable to take such a broad approach. Furthermore, many of the articles were of a largely descriptive nature: perceptions about MiP and attitudes towards ANC services are detailed, however, attempts at identifying the social and cultural factors that underlay these perceptions and attitudes were limited. This illustrates the need for further study of the themes highlighted in this review. Moreover, through employing a comparative, in-depth research approach it will be possible to move beyond describing perceptions, to investigate if, how and why such perceptions affect behaviour, and how these perceptions are formed and changed. Such in-depth understanding would provide a useful basis for the development of public health policy to reduce the burden of MiP.

The review has also identified specific topics that have been neglected in the qualitative research on MiP and that should be afforded particular consideration in further study. Adolescent pregnancy receives little attention in the literature, in spite of the vulnerability of this population group to MiP and the stigma that accompanies adolescent pregnancy in many societies and has implications for pregnancy care. Anaemia during pregnancy, its corresponding local disease categories and ideas about causation are also neglected topics, the study of which would contribute to understandings of MiP. The factors influencing gestational age at first formal ANC visit are also largely neglected in the reviewed literature, in spite of the impact that ANC attendance has on MiP interventions and on maternal health in general. Structural factors influencing the delivery and uptake of MiP intervention are also under-explored: the relationships and interactions between structural factors at a range of levels and how these influence behaviour are of particular interest.

Further research is also required in order to keep pace with the changing strategies of MiP prevention and control. For example, in terms of structural factors at the household level, research on decision-making regarding which family member(s) use ITNs is important, particularly in light of changing patterns of ITN distribution. Given the increased reliance on ACTs with varying treatment regimens, social scientists must also continue to explore related attitudes and understanding of new antimalarials. Moreover, as demonstrated by changes over time in understandings of the relationship between MiP and anaemia in Ghana [46], [60], knowledge, perceptions and beliefs rarely remain static. Social science research is therefore necessary to track such changes and explore the underlying social and cultural factors.

### Strengths and limitations of the review

This review provides a synthesis of a greater number of qualitative studies than the previous, albeit non-systematic, review focusing on social science research on MiP in Africa [17]. Searching the MiP Consortium Library ensured access to additional literature that would have been otherwise omitted from a search including only the conventional databases (OVID SP etc). The exclusion of non-English language research represents a limitation, however, as only five articles were excluded for this reason, the impact upon the themes identified in the review is likely to be minimal. The abstracts of the studies with unavailable full text were also assessed (see Appendix S2): several of the article were published over 15 years ago and therefore referred to outdated MiP interventions, others presented findings based on quantitative methods and those with relevant findings presented in the abstracts, largely confirmed the reviews findings (e.g. women lacking familiarity with IPTp [81] and adolescents not perceiving a greater risk of malaria during pregnancy [82]). It is unlikely therefore that the contents of these articles would have substantially altered the review's findings. Included studies were assessed as, on average, of a fair quality. This suggests that, in general, the findings are based on an overall sound body of research. However, the scope of the review's findings is restricted by the topics dealt with by the included articles.

### Conclusions

Qualitative methods have become increasingly prominent in research on the uptake and delivery of MiP interventions. Despite the increasing number of qualitative studies, to date, reviews of social science research on malaria prevention and control have identified a lack of translation of findings, particularly from qualitative research, into policy [18], [19], [29]. In light of this, the findings from this review represent a potentially useful resource for policy makers as well as researchers in the field of MiP.

The reviewed qualitative research has revealed a range of factors as relevant to the uptake of MiP interventions, which are important considerations for the design and implementation of strategies to reduce MiP-related morbidity and mortality. The following factors are of particular importance: malaria and MiP is often understood in terms of locally-defined illness categories that may incorporate other causal explanations in addition to mosquito bites; although pregnant women are viewed as particularly at risk of malaria infection, and MiP as having important health impacts, the vulnerability of pregnant women is sometimes considered in less-disease specific terms; ideas about the purpose of ANC services and health workers' attitudes and behaviour towards pregnant clients influence attendance at health facilities for ANC; concerns about specific MiP interventions are influenced by local discourses and the attitudes and behaviours of health staff; patterns of household decision-making and gender relations influence women's access to MiP interventions; shortages of health personnel and resources, and inadequate infrastructure and health worker training impact upon the provision of MiP interventions; and cost of services and distance to health facilities influence women's access to MiP interventions.

In spite of the recent increase in qualitative research on MiP and MiP interventions, the review has highlighted a range of topics that require further research and demonstrated the need to follow a comparative yet in-depth research strategy. Key areas requiring further research include: local disease categories and symptoms of MiP and anaemia during pregnancy; understandings of pregnant women's and adolescents' perceived vulnerability to malaria and its consequences; perceptions about MiP interventions; wider political discourses around interventions; and factors that influence ANC attendance, including timing of first visit. In response to this, as part of the MiP Consortium's Public Health Impact research theme, a programme of anthropological research employing qualitative methods has been developed and is currently being carried out. Research is being undertaken in four settings across sub-Saharan Africa (Ghana, Kenya and Malawi), to investigate comparatively the social and cultural context to MiP and the uptake of MiP interventions.

## Supporting Information

Appendix S1 **OVID SP and ISI Web of Knowledge databases.**
(DOC)

Appendix S2 **Articles with full text unavailable.**
(DOC)

Appendix S3 **Data extraction tables.**
(DOC)
