# Supplementary material for: Social and Cultural Factors Affecting Uptake of Interventions for Malaria in Pregnancy in Africa: A Systematic Review of the Qualitative Research
Source: PLoS One. 2011 Jul 20;6(7):e22452. doi: 10.1371/journal.pone.0022452 (PMC3140529; doi:10.1371/journal.pone.0022452)
Supplement: Appendix S1 — OVID SP and ISI Web of Knowledge databases. (DOC) [file pone.0022452.s001.doc]

### Appendix 1. OVID SP and ISI Web of Knowledge databases

| **OVID SP** | ISI Web of Knowledge |
| --- | --- |
| British Nursing Index and Archive (1985 – May 2010) | BIOSIS Previews® (1926 – May 2010) |
| Maternity and Infant Care (1971 – May 2010) | CABI (1973 – May 2010) |
| PsycINFO (1806 – May Week 3 2010) | Inspec® (1969 – May 2010) |
| Social Policy and Practice (April 2010) | MEDLINE® (1950 – May 2010) |
|  | Zoological Record® (1864 – May 2010) |
|  | Web of Science® (1899 – May 2010) ^‡^ |
|  | Current Contents Connect® (1998 – May 2010) |
|  | Derwent Innovations IndexSM (1980 – May 2010) |

^‡^ Web of Science includes the following databases: Science Citation Index Expanded (SCI-EXPANDED) (1899 – May 2010); Arts & Humanities Citation Index (A&HCI) (1975 – May 2010); Conference Proceedings Citation Index- Science (CPCI-S) (1990 – May 2010); Conference Proceedings Citation Index- Social Science & Humanities (CPCI-SSH) (1990 – May 2010)
