# Supplementary material for: Social and Cultural Factors Affecting Uptake of Interventions for Malaria in Pregnancy in Africa: A Systematic Review of the Qualitative Research
Source: PLoS One. 2011 Jul 20;6(7):e22452. doi: 10.1371/journal.pone.0022452 (PMC3140529; doi:10.1371/journal.pone.0022452)
Supplement: Appendix S2 — Articles with full text unavailable. (DOC) [file pone.0022452.s002.doc]

### Appendix 2. Articles with full text unavailable

| **Reference** | **Location** | **Type of publication** | **Comments** |
| --- | --- | --- | --- |
| [1] | Zambia | PhD thesis | Abstract accessed: qualitative data not mentioned. |
| [2] | Nigeria | Medical journal article | Abstract accessed: likely to be excluded based on the quantitative data collection |
| [3] | Malawi | Journal article | Abstract accessed: potentially interesting article that discusses the importance of qualitative data. However it is dated and discusses outdated interventions (chemoprophylaxis). |
| [4] | Malawi | Unknown | Abstract accessed: lack of familiarity with the purpose of IPTp with SP among women attending ANC. |
| [5] | Uganda | Medical journal article | Abstract accessed: adolescent girls did not consider themselves at risk of malaria when pregnant. Anaemia and low birth weight were not related to MiP. Malaria self-medication (with the counter drugs, herbs or both) was common. |
| [6] | Tanzania | Journal article | Abstract accessed: MiP identified by community members as an important health issue |
| [7] | Malawi | Medical journal article | Abstract accessed: explores cross-border access to MiP interventions: Mozambican women living in Malawi. |
| [8] | Nigeria | Tropical Medicine journal article | Abstract accessed: unclear and possibly relevant to MiP prevention and treatment for adolescents. However, the article is dated and potentially investigates outdated interventions. |
| [9] | Nigeria | Unknown | Based on title: likely to be excluded on other grounds (not focussed on MiP) but unclear |

# References

1. Asamoah W (2004) Assessment of the delivery of malaria control and PMTCT programmes to pregnant women attending antenatal services in Lusaka, Zambia. University of Liverpool.

2. Enato EFO, Mens PF, Okhamafe AO, Okpere EE, Pogoson E, et al. (2009) Plasmodium falciparum malaria in pregnancy: prevalence of peripheral parasitaemia, anaemia and malaria care-seeking behaviour among pregnant women attending two antenatal clinics in Edo State, Nigeria. Journal of Obstetrics and Gynaecology 29: 301-306.

3. Helitzer-Allen DL, Kendall C (1992) Explaining differences between qualitative and quantitative data: A study of chemoprophylaxis during pregnancy. Health Education Quarterly 19: 41-54.

4. Mahama IY (2004) Community based study of the coverage of intermittent preventive treatment amongst pregnant women in a rural area of southern Malawi.

5. Mbonye A, Neema S, Magnussen P (2006) Malaria in pregnancy, risk perceptions and care seeking practices among adolescents in Mukono district Uganda. Int J Adolesc Med Health 18: 561-573.

6. Mboera LEG, Kamugisha ML, Barongo V, Rumisha SF, Msangeni HA, et al. (2004) Community knowledge, perceptions and practices on malaria in Mpwapwa District, central Tanzania.

7. Muula A, Yiwombe Z, Matchaya M (2004) Accessibility of insecticide treated bed nets and intermittent presumptive treatment of malaria to Mozambicans obtaining healthcare in Southern Malawi: A case study of international disease control and equity in health.

8. Okonofua FE, Feyisetan BJ, Davies-Adetugbo A, et al. (1992) Influence of socioeconomic factors on the treatment and prevention of malaria in pregnant and non-pregnant adolescent girls in Nigeria.

9. Tekobo A, Tayo F, Mabadeje AF (2004) Knowledge and practice of drug retailers in malaria management in Lagos Nigeria: A preliminary survey.
