# Supplementary material for: Social and Cultural Factors Affecting Uptake of Interventions for Malaria in Pregnancy in Africa: A Systematic Review of the Qualitative Research
Source: PLoS One. 2011 Jul 20;6(7):e22452. doi: 10.1371/journal.pone.0022452 (PMC3140529; doi:10.1371/journal.pone.0022452)
Supplement: Appendix S3 — Data extraction tables. (DOC) [file pone.0022452.s003.doc]

Appendix 3: Data extraction tables

| Article | **Aim** | **Methods** ^a^ | Participants | **Country** | **Key findings from qualitative data** | **Grade** |
| --- | --- | --- | --- | --- | --- | --- |
| *Abdu, Z., et al., (2004.) “The impact of user fee exemption on service utilization and treatment seeking behaviour: the case of malaria in Sudan.” Int J Health Planning and Management* ***19****:S95-S106* | To assess the impact of different levels of user fee exemption on service utilization and treatment seeking behaviour for children under five years and pregnant women. | Experimental study design including: FGDs; IDIs (household surveys). | Mothers of children under five years (N=128 and 160); pregnant women (N=44 and 40); health workers. | Sudan | The qualitative data confirmed that under the fee exemption scheme pregnant women visited the clinic more often when they had malaria and were able to buy a full course of medication. | 29 |
| *Adhanom Ghebreyesus T., et al. (1996). “Community participation in malaria control in Tigray region in Ethiopia.” Acta Tropica* ***61****:145-156* | To describe the activities of the community based malaria control programme. | (Training and monitoring of CHW activities); FGDs. | Community members; CHWs. | Ethiopia | Pregnant women did not comply with weekly malaria chemoprophylaxis due to: a lack of knowledge about the reasons for and the safety of chemoprophylaxis; the belief that chemoprophylaxis during pregnancy causes abortion; lack of time to meet healthcare needs; and, the distance to the clinic. | NA |
| *Agyepong, I. A. & Manderson, L. (1994).”The diagnosis and management of fever at household level in the Greater Accra Region, Ghana.” Acta Tropica* ***58****:317-330* | To describe the folk diagnosis, etiology and management of malaria. | FGDs; IDIs; observations (cross-sectional survey). | Community members. | Ghana | Fever was the term predominately used for malaria. Mosquitoes were seen as playing role in malaria transmission via “drinking” dirty water. Adults treat their fever first at home with herbal remedies and other drugs, but pregnant women with fever wait a few days to see if it improves and if not go straight to the hospital because want to care for their pregnancy. Medicine in early pregnancy however “might spoil your pregnancy”. | 29 |
| *Agyepong, I. A., et al. (1997). "A comparative study of clinical and sociocultural aspects of anaemia among adolescent girls in rural Ghana." Acta Tropica* ***65****(3): 123-138.* | To investigate the prevalence of malaria and anaemia among adolescent girls, and community perceptions of blood, anaemia and malaria. | Participant observation; FGDs; IDIs; informal discussions; (structured interviews, blood examinations). | Adolescent girls (pregnant and non-pregnant); adult men; adult women; community leaders; local healers; parents/guardians of adolescent girls. | Ghana | MiP was not specifically associated with anaemia (locally conceptualised as bad blood/no blood in the body) though dizziness, weakness, weight loss and pallor were seen as symptoms. Possible underlying causes of anaemia in pregnancy were poor diet (including drugs) and anxieties such as being unmarried. | 28 |
| *Ahorlu, C.K., et al. (2007). “Children, Pregnant Women and the Culture of Malaria in Two Rural communities of Ghana.” Anthropology and Medicine* ***14****(2): 167-181* | “…[T]o elicit relevant contemporary ethnographic feature of malaria in children and pregnant women in two rural villages in Ghana for intervention”. | Free listing and rating; Participatory social mapping; IDIs; FGDs. | Community members; chiefs and elders (8 IDIs); women’s leaders (4 IDIs); pregnant women (8 IDIs); caretakers of children < five years (8 IDIs). | Ghana | Malaria was considered the most common health problem in the two communities. Malaria was grouped into a common fever or male fever – the former easily cured with herbs or medicines; the latter more difficult to cure. Pregnant women were not allowed to take medicines (neither herbal nor biomedical) without the advice of “experts” – at the clinic/hospital or local healers. Treatment at the clinics/hospitals was preferred but costs were described as prohibitive and higher than those of local healers. Malaria also caused reduction in blood. Respondents were unconvinced by the possibility or preventing malaria. Mosquito bites were amongst various causes of malaria. ITN were not used. | 29 |
| *Brabin, L., et al. (2009). “Rural Gambian women’s reliance on health workers to deliver sulphadoxine-pyrimethamine as recommended intermittent preventifve treatment for malari in pregnancy.” Malaria Journal* ***8****: 25* | “To assess whether rural Gambian women were aware of the importance of the timing of the two-dose IPT dose schedule and its relevance for drug safety”. | IDIs; FGDs. | Married women (8 FGDs); adolescent girls (3 FGDs); TBAs (1 FGD); men (4 FGDs) (41 IDIs). | The Gambia | Quickening not recognised as a specific stage in pregnancy. MiP known to cause anaemia and pre-term birth and to affect the foetus indirectly. Choloroquine identified as a drug to be avoided in early pregnancy due to bitter taste. TBAs encouraged women to visit ANC in first trimester. Women unclear about drugs they were given and accepted as safe if given by a nurse or doctor. There was some confusion about IPTp and iron treatments. Overall ignorance of IPTp schedule and its importance. Importance of Muslim understandings of foetal development. | 29.5 |
| *Chuma, J., et al., (2010). “Towards achieving the Abuja targets: identifying and addressing barriers to access and use of insecticide treated bednets among the poorest population in Kenya.” BMC Public Health* ***10****:137* | “[T]o explore barriers to ownership and use of ITNs among the poorest populations before and after the mass campaigns, to identify strategies for improving coverage, and to make recommendations on increased coverage levels can be sustained”. | FGDs (cross-sectional survey, semi-structured interviews). | Community members. | Kenya | Affordability seen as an important barrier to ITN ownership. Non-white ITNs were preferred. Community preferences were not considered in the design of ITNs. Pregnant women and children were recognised as being most at vulnerable from malaria but respondents were suspicious about the targeting of interventions at these groups – linked to concerns about demographic assault. ITNs should be available for all. Men control the household resources and therefore health-related decision-making. Subsidised ITNs intended for vulnerable groups could be obtained by anyone. Health workers admitting selling subsidised ITNs to top-up their salary. | 32 |
| *Deressa, W. & Ali, A. (2009). “Malaria-related perceptions and practices of women with children under the age of five years in rural Ethiopia.” BMC Public Health* ***9****:259* | “[T]o investigate the local perceptions, practices and treatment seeking behaviour among women with children under the age of five years”. | FGDs; IDIs (questionnaire survey). | Mothers of children under the age of five (3 FGDs, 5 IDIs). | Ethiopia | Respondents used the term Busaa to refer to malaria, which was considered a serious disease. Pregnant women, along with children were identified as especially vulnerable to malaria. The effects of MiP were well-articulated and prompt action recommended. Although malaria was linked to mosquito bites there were other factors associated. Reduced effectiveness of SP for treatment of malaria recognised. Lack of confidence in ITNs reported. | 33 |
| *Hassan, S. E. H., et al. (2008). "Retention and efficacy of long-lasting insecticide-treated nets distributed in Eastern Sudan: a two-step community-based study." Malaria Journal* ***7****(85): (20 May 2008).* | To investigate the retention of ITNs and their efficacy. | (Questionnaire survey); FGDs | Men; women (8 FGDs). | Sudan | Most FGD participants said they would like to have more ITNs than they had. People did not know if nets were impregnated or not, or needed to be, and were seen as for use during and after rainy season only. Some thought nets were too large. | 30 |
| *Kengeya-Kayondo, J. F., et al. (1994). "Recognition, treatment seeking behaviour and perception of cause of malaria among rural women in Uganda." Acta Tropica* ***58****(3/4): 267-273.* | To understand recognition, treatment seeking behaviour and perceptions of cause of malaria among rural women. | FGDs; IDIs; (semi-structured interviews) | Woman; women attending MCH clinic. (45 FGDs, 64 IDIs). | Uganda | Word used for malaria (omusujia) also refers to any kind of fever and generally feeling unwell. Seen to be caused by diet, environmental factors, mosquitoes and part of other illnesses. In pregnancy symptoms are miscarriage, vomiting, general weakness, heat in the stomach, coldness, joint pain, lack of appetite – seen as serious. Complications could also lead to loss of blood, miscarriage, turning yellow, mental health problems, few go to hospital first. | 25 |
| *Launiala, A., T. Kulmala, et al. (2006). "The importance of understanding the local context: women's perceptions and knowledge concerning malaria in pregnancy in rural Malawi." Acta Tropica* ***98****(2): 111-7.* | To discover how Yao women understand and explain MiP, how they perceive it, and what type of knowledge they have about it. | Two stage design: FGDs ; IDIs; drug identification exercises; participant observation; (KAP survey). | Women of reproductive age (n=34); traditional advisers (n=4); TBAs (n=2); traditional healer (n=1); man (n=1). | Malawi | No vernacular word for malaria or MiP. Malungo refers to various fevers aside from malaria. Mulungo in pregnancy, although a problem, is not seen as serious (anaemia, diarrhoea, STDs, AIDS, cholera and convulsions seen as more serious) and is common. There are two kinds of Malungo, one caused by mosquitoes, one by hard work. Concerns about Mulunogo were not related to pregnancy. Respondents did not know the effects of MiP. Mild fever during pregnancy was seen as normal, and can even indicate pregnancy. | 32.5 |
| *Launiala, A. and M. L. Honkasalo (2007). "Ethnographic study of factors influencing compliance to intermittent preventive treatment of malaria during pregnancy among Yao women in rural Malawi." Transactions of the Royal Society of Tropical Medicine and Hygiene* ***101****(10): 980-989.* | To examine pregnant women’s perceptions and knowledge about malaria medication and the use of ANC services. | Two stage design: FGDs ; IDIs; drug identification exercises; participant observation; (KAP survey). | Women of reproductive age (n=34); traditional advisers (n=4); TBAs (n=2); traditional healer (n=1); man (n=1). | Malawi | There were unclear messages about IPTp with SP from nurses relating to timing. There were shortages of SP and nurses demonstrated limited understanding of SP. Bitter tasting drugs were thought to cause miscarriage, yet most accepted SP during pregnancy, as doctors know what is best. SP was seen as a treatment rather than prevention for malaria (nets are for prevention). Timing of first ANC visit (average 24 weeks) prompted by desire to confirm pregnancy and it was common to wait until quickening - miscarriage was common and do not want the inconvenience of travelling a long distance to the clinic before being certain of the pregnancy). Mothers attended ANC to check that the baby was growing well and for fear of not being properly attended by nurses during delivery (e.g. by obtaining an ANC card). Health education was delivered in mother tongue of the nurses rather than that of the women. | 31 |
| *Mbonye, A. K., et al. (2006). "Perceptions on use of sulfadoxine-pyrimethamine in pregnancy and the policy implications for malaria control in Uganda." Health Policy* ***77****(3): 279-289.* | To asses perceptions of use of SP in pregnancy and identify policy implications. | FGDs; IDIs. | Adolescent girls (n=15); women 20-49 years (n=75); opinion leaders; local council officers; elderly midwives; retired female teachers; drug shop owners; TBAs; pregnant and non-pregnant women. | Uganda | Children seen as most at risk of malaria, followed by pregnant women due to poor diet and low bed net use. Men also saw women as vulnerable due to weak blood but primagravide and adolescents were not seen as at risk of malaria. Mild and severe malaria were distinguished. Fever, accompanied by lower abdominal and breast pain seen as normal after delivery. It was seen as not possible to have malaria without symptoms. SP viewed as treatment not prevention, and as strong enough to cause abortions and foetal abnormalities as health workers encouraged drinking of sweet fluids when taking SP. IPTp was also seen to cause drug resistance and it was better to use weaker drugs first. Perception of drug strength may be rooted in idea of ability to cure quickly, with weakness as a side effect. ANC services perceived as only supplying drugs and for obtaining the clinic card. | 30 |
| *Mbonye, A. K., et al. (2006). "Preventing malaria in pregnancy: a study of perceptions and policy implications in Mukono district, Uganda." Health Policy and Planning* ***21****(1): 17-26.* | To explore the perceptions and beliefs and practices associated with malaria prevention in pregnancy. | FGDs; IDIs. | Pregnant women; non-pregnant women; adolescent girls; men (10 FGDs); opinion leaders; local council officers; elderly midwives; retired female teachers; drug shop owners; TBAs; pregnant and non-pregnant women (40 IDIs). | Uganda | Malaria was seen as serious and pregnant women (and children) were seen as the most vulnerable. ITNs were not used because of their high cost and perception that the chemicals they contained were dangerous and could influence the outcome of a pregnancy. Adolescents, primigravidae, and men not seen as at risk. Adolescents do not use health facilities if pregnant because they try to abort due to the stigma associated with adolescent pregnancy. Cost and non-availability were constraints to ITN use, also chemicals and smell of repellents was reported to make women vomit. Women reluctant to buy ITN because this required money and if they spent money their husband would accuse them of have an affair with another man. Women attended ANC to get card, in case of problems during delivery. | 30 |
| *Mbonye, A. K., et al. (2006). "Treatment-seeking practices for malaria in pregnancy among rural women in Mukono District, Uganda." Journal of Biosocial Science* ***38****(2): 221-237.* | To assess perceptions of MiP, recognition of early signs or pregnancy and of malaria and cultural context of treatment seeking. | FGDs; IDIs. | Pregnant women; non-pregnant women; adolescent girls; men (10 FGDs); opinion leaders; local council officers; elderly midwives; retired female teachers; drug shop owners; TBAs; pregnant and non-pregnant women (40 IDIs). | Uganda | Omusujia (febrile illness) cited as pregnant women’s most common illness. All fever was said to be caused by mosquitoes or malaria, through the blurring of terminology. Pregnant women (and children) were thought to be at highest risk of malaria, which for pregnant women can result in miscarriage. In contrast, adolescents were not seen as being at risk of MiP. MiP was not associated with low birth weight. IPTp was relatively unknown. Anaemia was attributed to diet rather than malaria. | 29 |
| *Mbonye, A. K., et al. (2007). "Intermittent preventive treatment of malaria in pregnancy: evaluation of a new delivery approach and the policy implications for malaria control in Uganda." Health Policy* ***81****(2-3): 228-41.* | To assess the new approach of delivering IPTp with SP. | IDIs; (survey). | Pregnant women not participating in study (n=108); resource personnel; health workers; opinion leaders (n=60). | Uganda | Adolescents were not seen as vulnerable to malaria. Women that visited the health facilities were more likely to have greater knowledge of MiP and use bed nets more often. Women accepted IPT due to previous experience of MiP. Women that accessed IPT through TBAs, and drug store vendors trusted IPT because trusted them. The study enlisted support of husbands and this promoted acceptability. | 34 |
| *Mboney, A. K., et al. (2008). "Prevention and treatment practices and implications for malaria control in Mukono District Uganda." Journal of Biosocial Science* ***40****(2): 283-296.* | To asses the burden of malaria in relation to scaling up interventions. | Interviews; (household survey). | Opinion leaders (n=12); local council officials (n=20); community health workers (n=15); teachers (n=8); policemen (n=5); drug shop owners (N=23); pregnant women (n=22). | Uganda | Pregnant women (and children) were viewed as being most vulnerable to malaria. Drugs were kept for emergencies, and respondents self-medicate rather than going to the health facility, unless their illness gets worse. Price of drug more of a concern than dosage, side effects, expiry date. Expired drugs not linked to poor treatment of malaria. | 31 |
| *Mboney, A. K., et al. (2010). "Integration of malaria and HIV/AIDS prevention services through the private sector in Uganda." International Health 2: 52-58.* | To explore whether private midwives can provide prevention of mother-to-child transmission of HIV integrated with malaria prevention services in pregnancy and assess how this affects the access to and equity of services. | Interviews; (household survey, survey of private midwives’ clinics). | Civic leaders; HIV positive women; local council officials; midwives; teachers; pregnant women; non-pregnant women (n=66). | Uganda | Idea of integrated services unknown to pregnant and non-pregnant women and sceptical of ability of health facilities to provide multiple services. In public facilities, health staff complained of understaffing, lack of drugs and inadequate skills. Private midwives complained of a lack of skills, the high costs of drugs and a lack of supervision. Although health workers delivered IPTp, none did so in an integrated manner. | 29.5 |
| *Miaffo, C., et al. (2004). "Malaria and anaemia prevention in pregnant women of rural Burkina Faso." BMC Pregnancy and Childbirth* ***4****(18): (27 August 2004).* | To collect information on malaria and anaemia prevention behaviour amongst pregnant women. | FGDs; informant interviews; (questionnaire survey). | Pregnant women - both users and non-users of ANC; maternity health workers (n=4); TBAs (n=7); women’s group leaders (n=27). | Burkina Faso | Malaria and anaemia were seen as the most common problems during pregnancy. Malaria was viewed as preventable by chloroquine rather than nets. Most said they were satisfied with ANC quality. A small proportion knew services were free. ANC was therefore seen as costly. | 28 |
| *Mrisho, M., et al. (2009). “The use of antenatal and postnatal care: perspectives and experiences of women and health care providers in rural southern Tanzania.” BMC Pregnancy and Childbirth* ***9****:10* | To “…describe the perspectives and experiences of women and healthcare providers with regard to ANC and PNC in order to identify opportunities for improving maternal and newborn health services”. | IDIs; FGDs. | Women with children and pregnant women (8 IDIs, 8 FGDs); health care providers (8 IDIs). | Tanzania | Women perceived the administration of SP and the provision of ITNs as important parts of ANC services. ANC care was positively valued however for some women the only reason for attending ANC clinic was to obtain the health card that gives access to emergency assistance during delivery. | 31 |
| *Mubyazi, G., et al. (2005). "Intermittent preventive treatment of malaria during pregnancy: a qualitative study of knowledge, attitudes and practices of district health managers, antenatal care staff and pregnant women in Korogwe District, North-Eastern Tanzania." Malaria Journal* ***4****: 31.* | To assess the knowledge, attitudes and practices in relation to malaria control, especially IPTp. | IDIs; FGDs. | District Medical Officer (n=1); health service staff; Council Health Management Team (n=11); pregnant women. | Tanzania | Clinical officers reported that women are scared of side effects (including miscarriage) and may not swallow tablets. Different brand names seen as SP and not SP (even though both SP) and there were problems with provision e.g. clinics do not have clean water and cups. MiP among women leads to stillbirths, delivering a child with malaria, and too much bleeding during and after delivery, also persistent menstruation during pregnancy. Severe malaria was treated by traditional healers. Self-medication from drug stores was common. Reported negative experiences of ANC staff and services. Not all health staff understood IPTp. | 31 |
| *Mubyazi, G. M., et al. (2008). "Prospects, achievements, challenges and opportunities for scaling-up malaria chemoprevention in pregnancy in Tanzania: the perspective of national level officers." Malaria Journal* ***7****.* | To describe prospects, achievements, challenges and opportunities for implementing IPTp. | IDIs | National level malaria control officers (n=8). | Tanzania | Coverage, especially of 2^nd^ dose of IPTp is low. Problems of good monitoring of data. Late ANC attendance was reported to be a barrier for coverage; socio-cultural, economic factors contribute to this. Also, problem with the supply of SP, the effectiveness of DOT, poor skills and lack of knowledge of ANC staff, the reporting system, and problems with the health system more generally contributed to late ANC attendance. | 35 |
| *Mubyazi, G. M., et al. (2010). “Women’s experience and views about costs of seeking malaria chemoprevention and other antenatal services: a qualitative study from two districts in rural Tanzania” Malaria Journal* ***9****:54* | To describe the experience and perceptions of pregnant women about costs and cost barriers for accessing ANC services and emphasis on IPTp in rural Tanzania. | FGDs; (structured “exit interviews” post ANC). | Pregnant women; mothers with infants (24 FGDs). | Tanzania | High level of satisfaction with ANC services was reported. However complaints included: problems with transport; unnecessary referrals; varied users fees; unofficial payments and penalties; waiting times. The national ITN voucher scheme had encouraged women to visit ANC clinic. | 33 |
| *Mushi, A. K., et al. (2008). "Development of behaviour change communication strategy for a vaccination-linked malaria control tool in southern Tanzania." Malaria Journal* ***7****: 191.* | To describe the development of a behaviour communication strategy to support implementation of IPTi be health services. | FGDs; IDIs; (questionnaire survey). | Community workers; health workers; mothers of babies; pregnant women | Tanzania | Negative views of SP for pregnant women included adverse side effects, causing large babies (and therefore a more difficult delivery). A number of health workers had encountered pregnant women who were not willing to take SP. There were indications that women had taken SP in pregnancy without realising it. | 27 |
| *Mushi, A. K., et al. (2003). "Targeted subsidy for malaria control with treated nets using a discount voucher system in Tanzania." Health Policy & Planning* ***18****(2): 163-71.* | To evaluate a social marketing voucher scheme for bed nets. | FGDs; IDIs; (questionnaire survey). | Community leaders; parents of children under five; married and unmarried women (22 FGDs); MCH health staff; retail agents (4 IDIs). | Tanzania | Pregnant women were aware of the availability of bed nets. However other respondents demonstrated mixed awareness about scheme, how to take part, who the nets were for. And even discounted some could not afford. Also leakage. | 27 |
| *Ndyomugyenyi, Ret al. (1998). "The use of formal and informal services for antenatal care and malaria treatment in rural Uganda." Health Policy and Planning* ***13****(1): 94-102.* | To analyse reasons for use and non-use of ANC services and malaria prevention. | FGDs; IDIs; (structured questionnaire). | Pregnant women (10 FGDs); TBAs (4 IDIs); health workers (4 IDIs). | Uganda | Mothers attend ANC for examinations and for the ANC card. Some drugs, including chloroquine, are seen as causing abortion. Women complained of the cost and preferred to go to TBAs or neighbours. Health centres were lacking resources (staff and medicine). Formal health system was reported to be the last resort for treatment for malaria. Chloroquine had itching side-effect that deterred further use. Bitterness of drugs was also perceived by some as indicating that they should not be used during pregnancy. | 28 |
| *NetMark (2001). "NetMark formative qualitative research on insecticide treated materials (ITMs) in Nigeria."* | (Amongst others) To identify the factors that encourage and discourage: acquisition of nets; retreatment of nets with insecticide; and, use of treated nets by children under five and pregnant women. | IDIs; FGDs; observations. | Parents (or guardians) of children under five (51 IDIs) (10 FGDs); traders of insect control products (26 IDIs). | Nigeria | Children were seen as a higher priority for protection against malaria than pregnant women. There were concerns about the effects of insecticide treatments for ITNs on the health of pregnant women (and children) (through breathing in the chemicals) and related to sleeping under the nets – that this may results in miscarriage. | NA |
| *NetMark (2001). "NetMark formative qualitative research on insecticide treated materials (ITMs) in Senegal."* | (Amongst others) To identify the factors that encourage and discourage: acquisition of nets; retreatment of nets with insecticide; and, use of treated nets by children under five and pregnant women. | IDIs; FGDs. | Parents (or guardians) of children under five (51 IDIs) (10 FGDs). | Senegal | Some respondents recognised pregnant women as vulnerable to malaria (far more identified children). There were some concerns about the effects of retreating nets on pregnant women and the foetus – due to the smell of the product. | NA |
| *NetMark (2001). "NetMark formative qualitative research on insecticide treated materials (ITMs) in Uganda."* | (Amongst others) To identify the factors that encourage and discourage: acquisition of nets; retreatment of nets with insecticide; and, use of treated nets by children under five and pregnant women. | IDIs; FGDs; observations, | Parents (or guardians) of children under five (50 IDIs) (10 FGDs); traders of insect control products (32 IDIs). | Uganda | Pregnant women and children were viewed as being vulnerable to malaria (and therefore need special protection) – children more so than pregnant women. A minority of respondents linked malaria with miscarriages. Respondents were worried about the smell from mosquito coils and the harm this may cause to pregnant women. Although the concept of a mosquito was liked there were concerns about the effects of insecticide treatments for ITNs on the health of pregnant women and the foetus. | NA |
| *NetMark (2001). "NetMark formative qualitative research on insecticide treated materials (ITMs) in Zambia."* | (Amongst others) To identify the factors that encourage and discourage: acquisition of nets; retreatment of nets with insecticide; and, use of treated nets by children under five and pregnant women. | IDIs; FGDs; observations. | Parents (or guardians) of children under five (50 IDIs) (10 FGDs); traders of insect control products (25 IDIs). | Zambia | Pregnant women and children were identified as being especially vulnerable to malaria infection. Some respondents reported that treating the ITNs could be harmful for pregnant women. | NA |
| *Okrah, J., et al. (2002). “Community factors associated with malaria prevention by mosquito nets: an exploratory study in rural Burkina Faso.” Top Med Int Health* ***7****(3):240-248* | To examine malaria-related knowledge, attitudes and practices prior to the establishment of a ITN programme. | FGDs; IDIs; (questionnaire survey). | Men (5 FGDs) and women (5 FGDs) with a child less than five in the household; medical personnel; local tailors; ITN sellers; ITN users; drug sellers (9 IDIs). | Burkina Faso | No one equivalent term for malaria in any of the local languages. Using chloroquine for pregnant women was reported as a means of preventing malaria. Using an ITN and other means of preventing malaria were also reported. | 31 |
| *Smith, L. A. et al. (2010). “Intermittent screening and treatment versus intermittent preventive treatment of malaria in pregnancy: user acceptability.” Malaria Journal* ***9****: 18* | Assess the relative acceptability of Intermittent screening and treatment (IST) and IPTp. | FGDs. | Pregnant women who participated in the randomised controlled trial of IST versus IPTp. | Ghana | Women attended ANC clinic to discover if there were any problems with the pregnancy, particularly to ensure that the baby was correctly positioned. Although women mentioned various drugs that they received in the ANC clinic and they were familiar with some malaria drugs participants from different study arms did not refer to having received a different drug during the clinical trial. The differences were diluted by other aspects of ANC care. Blood tests, though painful were accepted provided the purpose of the test was known. Obtaining an ITN was one of the motives for attending ANC clinic. Health workers treated the pregnant women well and women trusted health workers. | 34 |
| *Soud, F. A. (2005). Medical pluralism and utilization of maternity health care services by Muslim women in Mombasa, Kenya, University of Florida****.*** | (Amongst others) “To describe the health care decisions made by Muslim women during pregnancy and to identify patterns of behavior in seeking prenatal health care”. | Ethnography; IDIs; (questionnaire survey). | Women attending ANC clinics; clinicians; Islamic healers; traditional healers; TBAs. Islamic religious leaders (Imams). | Kenya | Women identified malaria as a health problem during pregnancy. Women who suffered from malaria refused to be admitted to hospital for treatment because they had other children to care for. Women were aware of the dangers of not taking malaria prophylaxis during ANC visits. Women preferred western drugs to the available herbal remedies for malaria and bought these over the counter (chloroquine and Fanisdar) and self treated. Lack of anti-malarials for IPTp so not all women receive IPTp. | NA |
| *Tami, A., et al. (2006). "Use and misuse of a discount voucher scheme as a subsidy for insecticide-treated nets for malaria control in southern Tanzania." Health Policy Plan.* ***21****(1): 1-9.* | To estimate the extent to which subsidised nets reached the target communities (children under five and pregnant women). | FGDs; IDIs; (questionnaire survey). | Pregnant women and mothers with young children (5 FGDs); MCH staff (n=5). | Tanzania | Women received information from the MCH about the vouchers scheme – women in all FGDs had heard about the scheme but some had not seen vouchers. Health workers reported that vouchers could only be used to buy an ITN. | 29.5 |
| *Tolhurst, R., et al. (2008). "'I don't want all my babies to go to the grave': perceptions of preterm birth in Southern Malawi." Midwifery* ***24****:83-98* | To explore community understandings of preterm birth, infections during pregnancy and perinatal mortaility in Southern Malawi. | FGDs; critical incidence interviews; IDIs. | Mothers; fathers; grandmothers; (17 FGDs); women who have experienced preterm birth (n=11); health workers (n=5); TBAs (n=5): traditional healers (n=3). | Malawi | Of the “modern” illness categories, after sexually transmitted infections, malaria and anaemia were mentioned as causes of preterm birth. Preterm birth is also attributed to “traditional” illnesses (one of which could be malaria), witchcraft, impurity related to sexuality, death and blood, hard work, violence and the inappropriate use of medicine. It was generally agreed that bitter medicine, including SP, caused miscarriages and still birth. Some respondents argued that such medicine could be taken if prescribed. Though pregnant women are advised to go to ANC clinic, many buy drugs (including SP) from drug sellers. Women reported being chastised by health workers and cost as reasons for non-attendance. | 33 |
| *Waisa, P., et al. (2008). “ Acceptability of evidence-based neonatal care practices in rural Uganda – implications for programming.” BMC Pregnancy and Childbirth* ***8****:21* | To explore the acceptability of and barriers to the recommended evidence-based maternal and neonatal care and to home-visiting for maternal and neonatal care. | FGDs; IDIs. | Mothers less than 30 years (2 FGDs); mothers more than 30 years (4 FGDs); fathers (2 FGDs); childminders (2 FGDs); health workers (n=6); TBAs (n=4). | Uganda | ANC is literally referred to as “drinking medicine” and associated with being ill. Thus when women are ill and attend ANC they expect to receive a lot of medication. However women are frustrated when they receive on three SP tablets. Cost of transport prevented many women from attending ANC four times. Many diseases included high fever during pregnancy are treated first at home with herbs. | 31 |
| *Winch, P. J., A. et al. (1996). "Local terminology for febrile illnesses in Bagamoyo District, Tanzania and its impact on the design of a community-based malaria control programme." Social Science & Medicine* ***42****(7): 1057-1067.* | To examine local perceptions of malaria and malaria treatment practices, including local terminology. | Free listing; IDIs; FGDs; pile sorting; (questionnaire intervention). | Local officials; teachers; health workers. (40 IDIs). | Tanzania | Term perceived as corresponding closely to bio-medical definition of malaria (homa ya malaria), actually is not associated with severe anaemia or MiP, even though public health education uses this term. Diseases were divided into “our diseases” and “their diseases”, the latter which included malaria, which outsiders were expected to ask about. | 25 |

^a^Quantitative methods employed are listed in parantheses. IDI: in-depth interview. FGD: focus group discussion.
